# Electric Auditory Brainstem Response Audiometry in Cochlear Implants: New Recording Paradigm

**DOI:** 10.3390/audiolres14040049

**Published:** 2024-06-26

**Authors:** Takwa Gabr, Hossam Debis, Ahmed Hafez

**Affiliations:** 1 Audiovestibular Medicine Unit, Faculty of Medicine, Kafrelsheikh University, Elgeesh Street, Kafrelsheikh 33516, Egypt; 2 MED-EL GmbH, Cairo 11799, Egypt; hossam.debis@medel.com; 3 MED-EL GmbH, Riyadh 52091, Saudi Arabia

**Keywords:** electric ABR, cochlear implants, combined recording, channel interaction

## Abstract

(1) Background: Cochlear implants (CIs) are widely applied to recover audition for patients with severe degrees of or total hearing loss. Electrical stimulation using the electrically evoked ABR (E-ABR) can be recorded in CI recipients through the device. This work was designed to study E-ABR recorded individually from different channels located at the apical, middle, and basal cochlear regions in comparison to their simultaneous separated or adjacent combined recordings. (2) Methods: This study included 17 children fitted with unilateral cochlear implants. All children were subjected to impedance measurement, electrical compound action potentials (ECAP), and E-ABR recording of three channels located at the apical, middle, and basal cochlear regions. This was followed by simultaneous E-ABR recording of the three “separated” channels in comparison to E-ABR recording from three adjacent channels located at the middle cochlear region. (3) Results: Similar E-ABR latencies and amplitudes were found using either individual or simultaneously separated or adjacent combined recording. However, the mean amplitude measures of E-ABR for combined adjacent channels showed a positive correlation with the applied current level. (4) Conclusions: Combined E-ABR recording from adjacent channels is a faster and more reliable technique that can be used effectively without compromising the results of the recorded E-ABR.

## 1. Introduction

Cochlear implants (CIs) have been widely applied to recover hearing for patients with severe degrees of or total hearing loss. They use electrical pulses that can effectively stimulate the auditory pathways, allowing auditory perception [1].

Electrical stimulation using the electrically evoked ABR (E-ABR) can be recorded in CI recipients through the device. Within 10 ms of electrical stimulation, E-ABR can be captured from the scalp. Compared to the other E-ABR components, only waves III (eIII) and V (eV) are easily identified, whereas wave I (eI) is mostly obscured by stimulus artifacts [2]. The superior olivary nucleus and the inferior colliculus are assumed to be the neuronal generators of wave eIII and wave eV, respectively, as in the acoustic ABR, where E-ABR has two important parameters: latency and amplitude [3]. Generally, the absolute latencies of E-ABR are 1–2 ms earlier than the acoustic ABR due to the close proximity of the CI electrode to the spiral ganglion cells [4]. Regarding the amplitude, the eV peak-to-peak (V-VI) amplitude is small compared with other types of AEPs (0.2–0.5 µV) [5].

Clinically, E-ABR can be used pre-operatively to assess the surviving spiral ganglion cells (SGCs) through transtympanic promontory stimulation or direct stimulation of the round window [6]. This procedure objectively assesses the auditory pathway’s response to electric stimulation. Cases with cochlear pathology such as cochlear ossification are found to have higher E-ABR thresholds due to reduced SGCs and/or insufficient electric stimulus delivery to the cochlear nerve due to the ossification. Intraoperative E-ABR recording is performed after inserting electrode arrays into the cochlea to make sure that neural structures are responding appropriately before closing the wound [6].

Postoperatively, E-ABR serves different functions. First, it assesses the functionality of the cochlear nerve, which could differ according to the absence or presence of malformation (cochlear malformation, hypoplastic nerve, or auditory neuropathy). Eliciting E-ABR with good morphology indicates restoration of synchronized brainstem function with improved speech perception abilities [2]. Second, regular recording of E-ABR can be used to assess the improvement of the functionality of the auditory pathway as a result of plasticity in response to electrical stimulation over time. This can be used to evaluate the response of the neural tissue to stimulation via CI along different channels [7]. Additionally, E-ABR can be used in CI programming as an objective measure, especially in children where the E-ABR detection threshold was found to be between threshold (T) and comfortable (C) levels. Despite the fact that electric compound action potentials (ECAP) and the electric stapedial muscle reflex (ESRT) have more clinical value, E-ABR is preferable in some situations, such as auditory neuropathy, cases with questionable integrity of the auditory pathway through the brainstem, or some cochlear malformations where the ECAP cannot be recorded. Although the ESRT is more correlated with MCL, it could not be recorded in all cases due to increased middle ear stiffness in the implanted ear as a result of CI surgery or middle ear disorders in the non-implanted side [8]. Owing to the great flexibility in the stimulation and recording paradigms, E-ABR can be used as a suitable measure of the threshold (T) levels in CI cases [9,10,11]. Additionally, the amplitude growth function of E-ABRs has a strong correlation with neural loss. Therefore, E-ABR can be used as a measure of the auditory nerve fibers responding to electrical stimulation via CIs [12].

In most clinical settings, E-ABR recording is performed at individual channels in apical, middle, and basal electrode array regions to give a comprehensive idea of the performance of CIs across low-, mid-, and high-frequency regions [13]. This sounds inconvenient for young CI users who cannot sit quietly for a long time during single-channel E-ABR recording and may require sedation that might not be available in some CI centers.

Therefore, the idea of simultaneously recording E-ABR from the three channels at the three electrode array regions emerged in our minds. The mode of interaction among the three channels during simultaneous stimulation is still not clear, and it could impact E-ABR latencies or amplitude. This interaction could also be affected by the location of the stimulated electrodes, whether they are separated from each other or adjacent. Therefore, we hypothesized that simultaneous stimulation of E-ABR at three cochlear channels at a time would be less time consuming than individual recording. It will also allow neural evaluation along the whole channel (three at a time) in a time frame that is much shorter than that of the individual recording. Therefore, it will be more suitable for children.

Therefore, this work was designed to find a fast and reliable procedure for recording E-ABR, where E-ABR is recorded individually from three different channels located in the apical, middle, and basal cochlear regions, in comparison to their simultaneous recording. Additionally, simultaneous E-ABR recording from the selected three separate channels will then be compared with simultaneous E-ABR recording from another three adjacent channels located at the mid-frequency region of the cochlea.

## 2. Materials and Methods

### 2.1. Participants

All participants were recruited from the Audiovestibular Medicine Unit at Kafrelsheikh University Hospital in Egypt. Parental consent was obtained after the study protocol was explained. The study adhered to the ethical principles for medical research of the World Medical Association (Declaration of Helsinki) and had the approval of the institutional ethical committee (KFSIRB200-95).

The inclusion criteria included children and adolescents aged 8–18 years with pre- or peri-lingual sensorineural hearing loss (SNHL) who were fitted with a unilateral CI, had normal inner ear anatomy, regularly used the CI, performed appropriate device care, and had good aided response. Additionally, electrode array insertion that covered at least 1.5 cochlear turns (corresponding to a 540° insertion angle) with normal impedance measurements and confirmed normal array positioning within the cochlea was required, as validated by postoperative mastoid radiography. The exclusion criteria excluded children and adolescents who had a history of meningitis or head trauma, improperly used a CI, or had experienced repeated CI maintenance issues.

### 2.2. Electrophysiological Assessment and Stimulation Protocol

Participants underwent a series of tests, including impedance measurement, ECAP, and electrically evoked ABR recording.

For E-ABR stimulation, the MAESTRO system software was used (version 9.0.3, MED-EL, Innsbruck, Austria). For stimulation, biphasic current pulses of 25 µs/phase were used. Initially, at each selected channel (in both individual and combined recordings), participants were asked to rate the level of the perceived pulse to determine the most comfortable level (MCL). Subsequently, a series of biphasic pulses was delivered in monopolar stimulation mode at a rate of 34 Hz at the MCL.

Electrically evoked potential responses were recorded using Navigator Pro (Biologic EP317, version 5.46, Seyssinet-Priset, France). A triggering cable was used to synchronize the stimulation and the recording systems. The electrode montage followed the International 10–20 System, with the active electrode placed on the forehead (FPz), the reference electrode placed on the contralateral mastoid, and the ground electrode placed on the back of the neck.

Two to three runs of 1000 sweeps were acquired for the two E-ABR recordings. Excessive noise was eliminated when needed through artifact rejection and with a band-pass filter with cutoff values of 0.3–3 kHz.

#### 2.2.1. Individual Channel Stimulation

Three individual channels were used for stimulation, representing three different cochlear regions: apical (channel 2, C2), middle (channel 6, C6), and basal (channel 10, C10). For each channel, initial stimulation was performed at the MCL after the patient was asked to rate the presented current level, to avoid any discomfort. The current intensity was then reduced in steps of 20 cu. When approaching the E-ABR threshold level, smaller step sizes of 3–5 units were used. The threshold was defined as the lowest current level that could elicit wave V with a 0.1 µV amplitude in two replications of the stimulus condition [13]. At each channel, wave V latency and amplitude were calculated at the threshold level. For combined stimulation, the same procedure for individual stimulation was employed, where stimulation started after the MCL was set as before and descended in CL until it reached the E-ABR threshold level (wave V with 0.1 µV amplitude in two replicas of the combined stimulus condition), where both latencies and amplitudes were calculated.

#### 2.2.2. Combined Channel Stimulation

Two different combined stimulation configurations were tested: combined–separate and combined–adjacent. For combined–separate stimulation, all three channels that were used for individual stimulation were simultaneously stimulated at the threshold level. For combined–adjacent stimulation, three adjacent channels from the middle cochlear region (C5, C6, and C7) were stimulated at the threshold level. In both combined stimulation settings, if robust wave V responses were recorded at the threshold level determined from individual channel stimulation, the current level was reduced until the threshold level with combined stimulation was reached. With all combined stimulation configurations, both the latency and amplitude of wave V were calculated at the threshold level.

Statistical analysis was performed using R software version 4.2.2 (1 November 2022). Descriptive statistics were calculated for quantitative data, including mean, standard deviation (SD), and range, while absolute values and percentages were used for qualitative categorical variables. Latency in milliseconds (ms) and amplitude in microvolts (µV) were compared between apical, middle, and basal channels, as well as between combined–separated and combined–adjacent channels. Effects were compared using repeated-measures ANOVA with the Bonferroni correction. Pairwise comparisons were made using the paired *t*-test and the Wilcoxon signed-rank test. The normality assumptions were checked using the Shapiro–Wilk test, and *p* values ≤ 0.05 were considered significant.

## 3. Results

A post hoc study power analysis was performed using the Generalized Eta Squared (GES, η^2^) as an effect size for repeated-measures ANOVA designed for latency and amplitude as study outcomes. For the latency variance between apical, middle, and basal channels in individual stimulation, the GES (η^2^) equaled 0.99; therefore, the achieved power (1 − β) at the 5% level of significance with a sample size of 17 ears was approximately 1.0. Also, between adjacent and separated channels in combined stimulation, the GES (η^2^) equaled 0.99, and the achieved power was approximately 1.0, while, for the amplitude variance between apical, middle, and basal channels in individual stimulation, the GES (η^2^) equaled 0.48; therefore, the achieved power (1 − β) at the 5% level of significance with a sample size of 17 ears was approximately 0.86, and, between adjacent and separated channels in combined stimulation, the GES (η^2^) equaled 0.73, and the achieved power was approximately 0.98, indicating that a sample size of 17 ears was sufficient to achieve at least 0.86 power. The Generalized Eta Squared for repeated measures and the post hoc power analysis were calculated using packages (“ez” and “pwr”) in R software version 4.2.2.

This study included 17 children and adolescents (12 male, 5 female) who had been unilaterally fitted with a CI. The etiology of hearing loss was hereditary in 12 participants, post-febrile in 4, and idiopathic in 4. Their mean age was 11.7 ± 3.3 years old (range of 8–18 years). Their mean age at implantation was 3.2 ± 1.2 years. Regarding the inserted electrode types, seven cases received the STANDARD type (41.2%), six cases received FLEX28 (35.3%), and four cases received FORM24 (23.5%).

### 3.1. E-ABR with Individual Stimulation

**Figure 1:**
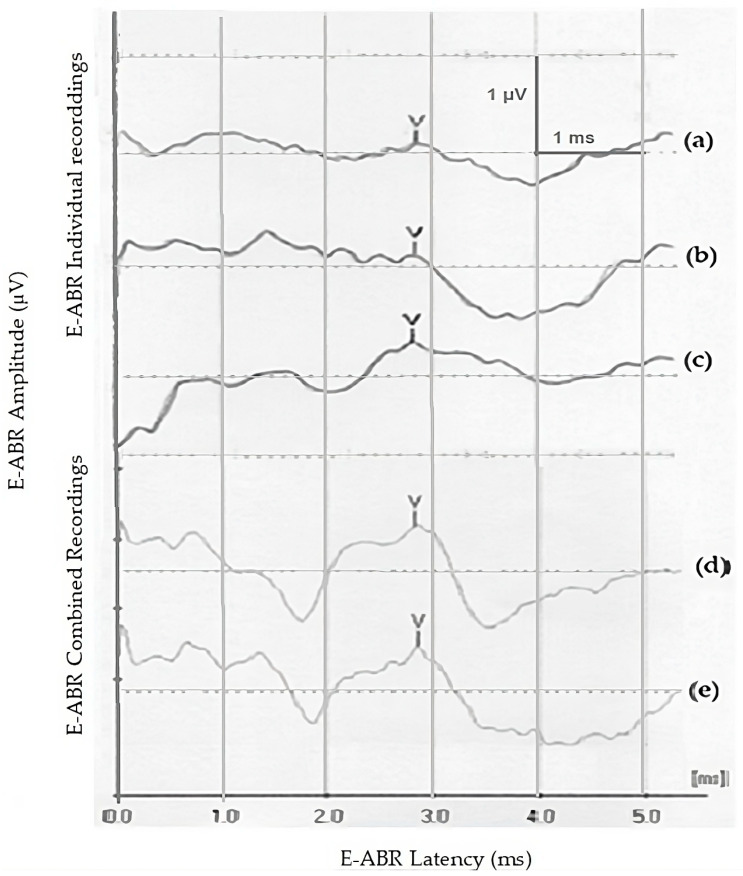
Individual E-ABR recording: C2 (**a**), C6 (**b**), and C10 (**c**); and combined E-ABR recording: separated (**d**) and adjacent (**e**).

**Figure 2:**
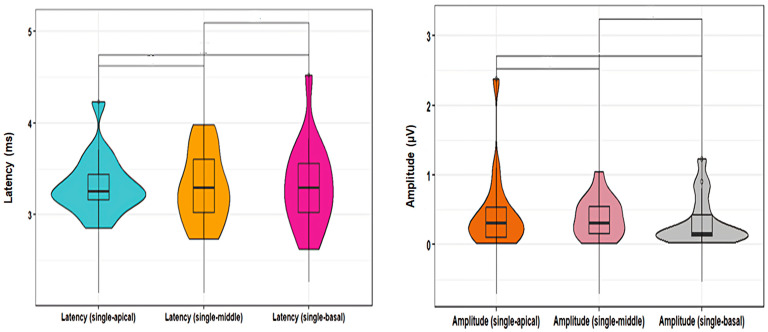
Comparison between E-ABR wave V latencies and amplitudes recorded at the apical, middle, and basal channels.

Example waveforms from individual stimulation at the three cochlear locations are provided in Figure 1. In individual stimulation, the mean current level (± SD) used for stimulation at the apical, middle, and basal channels was 347.03 ± 44.5, 373.53 ± 25.2, and 370 ± 58.2 µA, respectively, with the mean current level being 364.7 ± 84.3 µA. The ANOVA test showed that the mean latency of wave V at the apical, middle, and basal channels was not significantly different (family-wise *p* = 0.97; pairwise *p* values = 1 for each pair with Bonferroni adjustment for *t*-test) (Table 1; Figure 1). Similar non-significant findings were also found regarding the mean amplitude of wave V at the three channels (family-wise *p* = 0.66; *p* values = 1 for each pair with Bonferroni pairwise adjustment for Wilcoxon test) (Table 1; Figure 2).

### 3.2. E-ABR with Combined Stimulation

**Figure 3:**
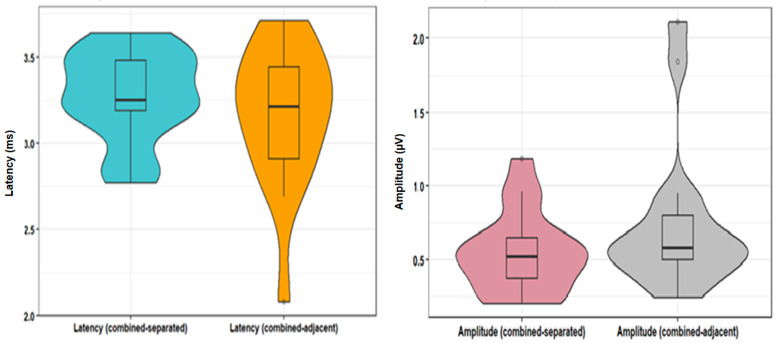
Comparison between E-ABR wave V latencies and amplitudes recorded during combined–separated and combined–adjacent recordings.

**Figure 4:**
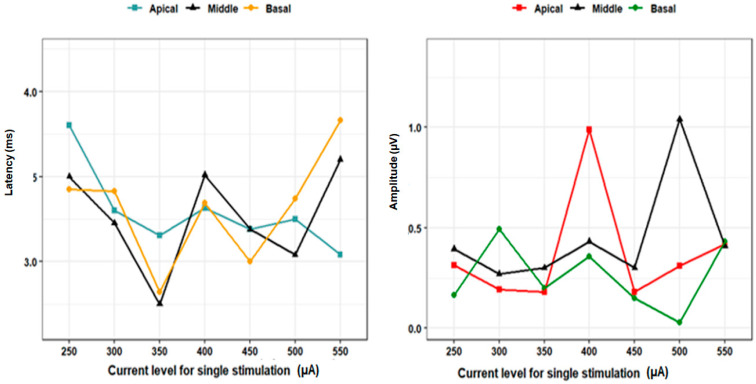
Current level and E-ABR estimates of latency and amplitudes for single stimulation.

Example waveforms from both configurations of combined stimulation are provided in Figure 1d (separated) and Figure 1e (adjacent). In combined–separate stimulation, the average current level was 207.4 ± 43.1 µA, the mean latency of wave V was 3.3 ± 0.3 ms, and the mean amplitude was 0.74 ± 0.26 µV. In combined–adjacent stimulation, the average current level was 200.0 ± 43.3 µA, the mean latency of wave V was 3.2 ± 0.4 ms, and the mean amplitude was 0.54 ± 0.14 µV. Paired *t*-test and Wilcoxon signed-rank test showed that neither the latencies (t = 0.117, *p* = 0.82, df = 16) nor amplitudes (t = 194, *p* = 0.088, df = 16) differed significantly between combined–separate and combined–adjacent stimulation, respectively (Table 2; Figure 3 and Figure 4).

### 3.3. Comparisons between Individual and Combined Stimulation

Multiple pairwise comparisons between individual stimulation and both configurations of combined stimulation revealed no significant differences. A series of Paired-Sample *t*-tests or Wilcoxon signed-rank tests were applied in this comparative analysis according to the normality assumptions of the measurements. Therefore, data with similar observations were grouped together into samples 1 and 2 and compared. For example, latency data obtained from the individual apical channels were compared with the latencies of the individual middle channels, then compared with the individual basal channels. The same comparison was performed between combined–separated and combined–adjacent channels, which were then compared with individual recordings at the apical, middle, and basal channels. The *p* value was adjusted using the Bonferroni adjustment methods for each comparison to avoid test type I (alpha) errors within the obtained results. This comparison was performed for wave V latency (Table 3) and amplitude (Table 4). The calculation of the wave V latency difference between different samples was performed. The difference between samples 1 and 2 in individual recordings revealed a negligible difference. The wave V latency difference between the combined–separated and combined–adjacent recordings was 0.11 ms, which was non-significant. The wave V latency difference between individual and combined (either separated or adjacent) recordings revealed a difference that ranged between 0.02 ms (between combined–separated and individual middle) and 0.16 ms (between combined–adjacent and individual apical and also between combined–adjacent and individual basal channels).

The wave V amplitude difference between samples 1 and 2 in individual recordings was 0.069 µV and 0.131 µV, respectively, which was non-significant. The wave V amplitude difference between combined–separated and combined–adjacent recordings was 0.194 µV (higher in combined–adjacent); however, it was not significant. The amplitude difference between individual and combined (either separated or adjacent) recordings revealed that the combined–adjacent recordings were higher than the three individual recordings; however, they did not reach the significant level after adjustment of the *p* value (to avoid type I (alpha) errors, as mentioned before).

### 3.4. Current Level and E-ABR Estimates

**Figure 5:**
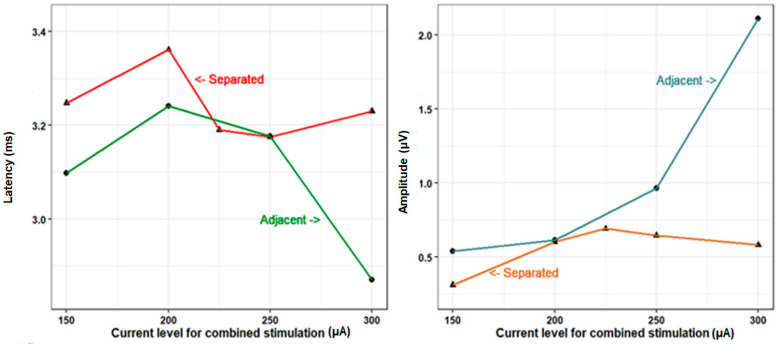
Current level and E-ABR estimates of latency and amplitudes for combined stimulation.

The latency and amplitude of wave V were studied as a function of increasing current levels with both individual and combined stimulation. With individual stimulation, no clear relationship was observed between increasing current level and latency or amplitude (especially at the middle and basal channels) (Figure 5). With both combined–separate and combined–adjacent stimulation, no clear relationship was observed between increasing current level and latency; however, a trend towards increasing amplitude with higher current levels was observed with combined–adjacent stimulation (Figure 5). However, the current level used for individual stimulation (364.7 ± 84.3 µA) was higher than that used for combined–separated stimulation (207.4 ± 43.1 µA) or combined–adjacent stimulation (200.0 ± 43.3 µA).

## 4. Discussion

E-ABR recordings are objective and effective methods for evaluating the conduction functions of the ascending auditory pathway up to the level of the brainstem [14]. The ability to evoke E-ABRs correlates with the number of surviving cells within the spiral ganglion [15]. Pre-operatively, E-ABRs can be used to determine the extent of the excitability of the auditory nerve and brainstem of a CI candidate. They can also be used to predict the functional outcomes of electrical stimulation of the auditory nerve [16]. Postoperatively, E-ABRs can be used to monitor the functionality of the implant and to verify stimulation of the auditory pathway [17].

In this study, 17 unilateral CI users were recruited. All were fitted with implants and audio processors from one manufacturer. In the first part of this study, E-ABRs were elicited from single-channel stimulation at three individual channels located at the apical, middle, and basal cochlear regions. Robust wave V responses were recorded from all sites in all cases with relatively shorter latencies than those reported in the literature (e.g., [18]). This could be due to the early maturation of both the upper and lower auditory brainstem as well as the auditory nerve as a result of early stimulation with a CI. Gordon et al. (2016) [2] reported that wave III and V of the E-ABR showed a reduction in latencies during the first year of CI use. Our results showed that both E-ABR latencies and E-ABR amplitudes were similar across the three elicitation sites. Similar findings were reported by Danieli et al. [19]. However, different results were reported by Thai-Van et al. [20] and Said and Afifi [18], who observed that E-ABRs recorded from apical channels had steeper and earlier responses than those recorded from the basal channels.

For combined stimulation, two configurations were used: combined–separate and combined–adjacent. Stimulation was performed at threshold levels of 207.4 ± 43 µA and 200.0 ± 43.3 µA, respectively, which was much lower than the single recording (364.7 ± 84.3 µA). Monopolar stimulation was used in this study. This mode is known to produce a relatively broad spread of excitation, which increases the potential for channel interactions [21]. A general principle in electrical stimulation is to ensure the patients’ safety by keeping the current level at a comfortable level. This is mandatory in CIs since the majority of patients are children. Electrical charge is the byproduct of both current intensity and pulse width. Therefore, the proper selection of these two parameters is very important in stimulating the neural structure through the CI. Additionally, the stimulus artifact amplitude related to the activation of surrounding tissue would be lower with a reduction in the current level, leading to better recording accuracy [22,23]. This study showed that the use of combined stimulation may permit the elicitation of E-ABRs at lower stimulation levels without compromising the response or causing patient discomfort.

Regarding the E-ABR latencies, both types of combined recording had similar wave V latencies. The amplitude of wave V was higher in the combined–separate recording (0.74 ± 0.5 µV) than in the combined–adjacent recording (0.54 ± 0.28 µV), although this difference was not significant. This finding emphasizes the possibility of spatial channel interaction during E-ABR recording in adjacent channels. Theoretically, each individual cochlear electrode should stimulate a certain neural population to provide an independent channel of information. However, the situation is different in practice due to broad spatial neural excitation with subsequent overlap of activation, which occurs as a result of the conductive environment of the cochlea [24]. Stimulation of the neural population that falls in the overlapping region of the adjacent channels may fire in response to the stimulation of the first channel and might be unlikely to fire again during stimulation of the other two adjacent channels due to the absolute refractory period of the auditory neurons [25]. Additionally, the physical interactions between two or more stimulated channels can occur due to electric field interactions. This depends on the amplitude and duration of the electrical pulses [23,26] as well as the electrode configuration and impedance [27,28]. In this study, channel interactions in combined E-ABR recording appeared in two situations. The first occurred when a lower level of current was used to elicit E-ABR wave V at the threshold in the combined recordings (200 and 207 cu) compared with 364 cu in a single recording. The second occurred when a higher amplitude of wave V was observed in combined–separate recording than in combined–adjacent recording.

In this study, we observed that increasing the current level had no significant effect on either E-ABR latencies or amplitudes for individual channel stimulation at apical, middle, and basal sites. However, the apical channels showed more consistent latencies with the least fluctuation when compared with the other channels. Regarding the amplitude, the basal channel again showed more consistent values with increasing current level (Figure 4).

Regarding the combined recordings, there was a tendency for the combined–separate recording to be shorter in latencies and higher in amplitude with increasing current level > 250 µA (Figure 5). This could be explained by the spread of excitation during electrical stimulation and the possibility of channel interaction that increases with increasing current levels due to the spread of electrical current to neurons beyond those that directly face the stimulated channels. This potentially causes overlapping of the neighboring frequency channels depending on the amplitude of the electric field and its diffusion through the cochlear fluid [29].

The current results indicate that combined E-ABR recording is of clinical importance to CI users. Its clinical value was emphasized in assessing neural tissue integrity in those individuals through a more rapid and consistent procedure. Combined E-ABR can be evoked at a much lower stimulation level than individual recording (≈200 µA for combined recording versus 364.7 µA for single recording) with good morphology and quality of recording. Additionally, the E-ABR amplitude for combined–separated recording was higher than that of combined–adjacent. This has the advantage of recording E-ABR at much lower current level with good morphology from the neural tissue in different regions (apical, middle, and basal) in a more comfortable and rapid procedure. This is very important when evaluating cases where time and comfort are essential, as in children.

The relatively small sample size and the lack of pre-operative E-ABR data for unilateral CI patients were considered study limitations. This might affect the data interpretation, especially in the context of comparison with pre-operative E-ABR recordings.

## 5. Conclusions

Generally, E-ABR recordings using combined stimulation provide good morphology, higher amplitudes, and reliable responses within a quite reasonable recording time. Additionally, they are elicited with much lower current levels than individual stimulation, making them more comfortable to many CI users, especially children. Specifically, E-ABR recording using combined–separate stimulation provides higher E-ABR amplitude, making it more advantageous than the combined–adjacent stimulation. This is due to the less likely occurrence of channel interactions during stimulation. This makes the combined–separate stimulation more suitable for assessing the neural tissue at different cochlear partitions in a rapid and more comfortable way.

## Figures and Tables

**Table 1:** E-ABR latencies and amplitudes at different channel locations within the cochlea.

	Individual Channel Recording					
Current Level	Latency (ms)			Amplitude (µV)		
364.7 ± 84.3 (Range: 250–550)	Apical Cs	Middle Cs	Basal Cs	Apical Cs	Middle Cs	Basal Cs
	Mean ± SD	Mean ± SD	Mean ± SD	Mean ± SD	Mean ± SD	Mean ± SD
	Min–Max	Min–Max	Min–Max	Min–Max	Min–Max	Min–Max
	3.32 ± 0.3	3.29 ± 0.4	3.31 ± 0.5	0.5 ± 0.6	0.4 ± 0.3	0.3 ± 0.3
	2.85–4.23	2.73–3.98	2.78–3.98	0.02–2.37	0.01–1.04	0.03–1.23
	*p* = 0.97			*p* = 0.66		

Cs: channels.

**Table 2:** Comparison of E-ABR latencies and amplitudes between the combined–separated and combined–adjacent channels.

	Combined–Separated Recordings Mean ± SD Min–Max	Combined–Adjacent Recordings Mean ± SD Min–Max	*t*-Test
Current level	207.4 ± 43.1	200.0 ± 43.3	
	150–300	150–300	
Latency (ms)	3.3 ± 0.3	3.2 ± 0.4	t = 0.117
	2.77–3.64	2.08–3.71	*p* = 0.82
Amplitude (µV)	0.74 ± 0.26	0.54 ± 0.14	t = 194
	0.20–1.18	0.24–2.11	*p* = 0.088

**Table 3:** Multiple comparative analysis of wave V latency measurements from individual stimulation, combined stimulation of separate channels, and combined stimulation of adjacent channels.

Sample 1	Sample 2	Sample 1	Sample 2	Absolute Mean Difference	Adj. *p* Value ^(1)^
		Mean ± SD	Mean ± SD		
Individual apical	Individual middle	3.32 ± 0.33	3.29 ± 0.39	0.03	1.0 ns
Individual apical	Individual basal	3.32 ± 0.33	3.31 ± 0.47	0.00	1.0 ns
Individual middle	Individual basal	3.29 ± 0.39	3.31 ± 0.47	0.03	1.0 ns
Combined separated	Combined adjacent	3.27 ± 0.26	3.17 ± 0.41	0.11	1.0 ns
Combined separated	Individual apical	3.27 ± 0.26	3.32 ± 0.33	0.04	1.0 ns
Combined adjacent	Individual apical	3.17 ± 0.40	3.32 ± 0.33	0.16	1.0 ns
Combined separated	Individual middle	3.27 ± 0.26	3.29 ± 0.39	0.02	1.0 ns
Combined adjacent	Individual middle	3.16 ± 0.41	3.29 ± 0.39	0.13	1.0 ns
Combined separated	Individual basal	3.27 ± 0.26	3.31 ± 0.47	0.04	1.0 ns
Combined adjacent	Individual basal	3.16 ± 0.41	3.31 ± 0.47	0.16	1.0 ns

^(1)^: for Bonferroni-adjusted pairwise paired *t*-test, ns: non-significant difference (significance if *p* ≤ 0.05).

**Table 4:** Multiple comparative analyses of wave V amplitude measurements from individual stimulation, combined stimulation of separate channels, and combined stimulation of adjacent channels.

Sample 1	Sample 2	Sample 1	Sample 2	Absolute Mean Difference	Adj. *p* Value
		Mean ± SD	Mean ± SD		
Individual apical	Individual middle	0.46 ± 0.57	0.39 ± 0.29	0.069	1.0 ^(1)^ ns
Individual apical	Individual basal	0.46 ± 0.57	0.33 ± 0.35	0.131	1.0 ^(1)^ ns
Individual middle	Individual basal	0.39 ± 0.29	0.33 ± 0.35	0.062	1.0 ^(1)^ ns
Combined separated	Combined adjacent	0.74 ± 0.50	0.74 ± 0.50	0.194	1.0 ^(1)^ ns
Combined separated	Individual apical	0.74 ± 0.50	0.46 ± 0.57	0.086	1.0 ^(1)^ ns
Combined adjacent	Individual apical	0.55 ± 0.28	0.46 ± 0.57	0.280 *	0.232 ^(1)^ ns
Combined separated	Individual middle	0.74 ± 0.50	0.39 ± 0.29	0.155	1.0 ^(2)^ ns
Combined adjacent	Individual middle	0.55 ± 0.28	0.39 ± 0.29	0.349 *	0.231 ^(1)^ ns
Combined separated	Individual basal	0.74 ± 0.50	0.33 ± 0.35	0.218 *	0.331 ^(1)^ ns
Combined adjacent	Individual basal	0.55 ± 0.28	0.33 ± 0.35	0.412 *	0.150 ^(1)^ ns

^(1)^: for Bonferroni-adjusted pairwise Wilcoxon signed-rank test, ^(2)^: for Bonferroni-adjusted pairwise paired *t*-test, *: significant before adjustment. *p* < 0.05 is significant.

## Data Availability

Data are unavailable due to privacy and ethical restrictions.
